# Combined Organ Transplantation in Patients with Advanced Liver Disease

**DOI:** 10.1055/s-0044-1788674

**Published:** 2024-07-25

**Authors:** Ingrid Wei Zhang, Isabella Lurje, Georg Lurje, Christoph Knosalla, Felix Schoenrath, Frank Tacke, Cornelius Engelmann

**Affiliations:** 1 Department of Hepatology and Gastroenterology, Charité Universitätsmedizin Berlin, Berlin, Germany; 2 Berlin Institute of Health (BIH) at Charité - Universitätsmedizin, Berlin, Germany; 3 European Foundation for the Study of Chronic Liver Failure (EF CLIF) and Grifols Chair, Barcelona, Spain; 4 Department of Surgery, Charité Universitätsmedizin Berlin, Berlin, Germany; 5 Department of Cardiothoracic and Vascular Surgery, Deutsches Herzzentrum der Charité, Berlin, Germany; 6 Charité - Universitätsmedizin Berlin, Corporate Member of Freie Universität Berlin and Humboldt-Universität zu Berlin, Berlin, Germany; 7 DZHK (German Centre for Cardiovascular Research), Partner Site Berlin, Berlin, Germany

**Keywords:** liver transplantation, combined transplantation, allocation, immunotolerance, machine perfusion

## Abstract

Transplantation of the liver in combination with other organs is an increasingly performed procedure. Over the years, continuous improvement in survival could be realized through careful patient selection and refined organ preservation techniques, in spite of the challenges posed by aging recipients and donors, as well as the increased use of steatotic liver grafts. Herein, we revisit the epidemiology, allocation policies in different transplant zones, indications, and outcomes with regard to simultaneous organ transplants involving the liver, that is combined heart–liver, liver–lung, liver–kidney, and multivisceral transplantation. We address challenges surrounding combined organ transplantation such as equity, utility, and logistics of dual organ implantation, but also advantages that come along with combined transplantation, thereby focusing on molecular mechanisms underlying immunoprotection provided by the liver to the other allografts. In addition, the current standing and knowledge of machine perfusion in combined organ transplantation, mostly based on center experience, will be reviewed. Notwithstanding all the technical advances, shortage of organs, and the lack of universal eligibility criteria for certain multi-organ combinations are hurdles that need to be tackled in the future.

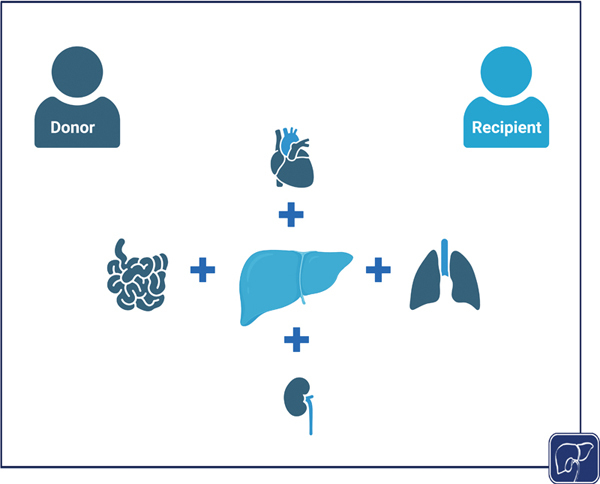

Over the past two decades, combined transplantation of heart–liver (CHLT), liver–lung (CLLT), and liver–kidney (CLKT) is an increasingly performed procedure worldwide despite the persistent challenge of organ donor shortage ( Fig. 1 ). Waitlist mortality is higher in patients listed for CHLT and CLLT, and multi-organ transplant (MOT) can be life-saving in selected patients with end-stage liver disease (ESLD) and concomitant dysfunction or failure of another organ, who otherwise would not survive. 1 The technical advances such as refinement of operation techniques, normothermic machine perfusion (NMP), and improved posttransplant management of patients have contributed to the success of MOT. Despite their growing importance, the number of MOT per health care center remains low, and there is a scarcity of results from human trials.

**Fig. 1 Number of performed transplants per year for different organ combinations in the United States and the Eurotransplant zone from 2003 to 2023.**
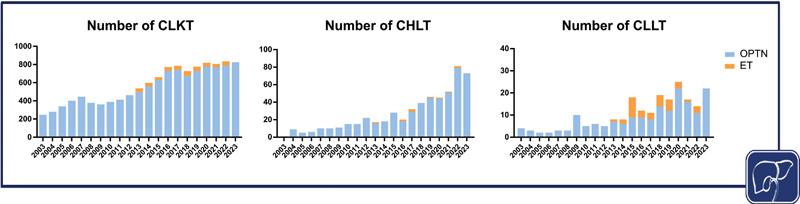
The data were extracted from the United Network for Organ Sharing Data and Transplant Statistics and the Eurotransplant Statistics Report Library. Created with BioRender.com. CHLT, combined heart–liver transplantation; CLKT, combined liver–kidney transplantation; CLLT, combined liver–lung transplantation.

Notwithstanding, ethical considerations of current practices regarding allocation policy, utility, equity, and futility remain and need to be addressed, even though there might not be a simple and straightforward solution. MOT has obvious advantages such as circumvention of immunological sensitization which might occur with serial surgeries and immunological protection to other allografts provided by the liver. However, in light of organ scarcity, the allocation priority given to MOT might divert organs away from single-organ transplant candidates. Efforts have been directed towards enhancing utility, for example by the introduction of the kidney-after-liver safety net in the United States in 2017, which theoretically allows living kidney donation. The policy implementation was able to interrupt the continuous rise in CLKT without affecting posttransplant outcomes of patients. 2 3

Transplant centers in the United States are required to submit data on outcomes of single-organ transplants, whereas MOTs are not included in this data reporting. In the Eurotransplant zone, submission of recipient outcome data to the Eurotransplant Registry by the transplant centers is, although strongly encouraged, on a voluntary basis, since Eurotransplant does not have the legal authority to compel return of these data. 4 This entails little published or skewed data on MOT allocation and outcomes, which makes identification and amelioration of listing criteria and analysis of graft survival challenging.

In the following, we will discuss the most frequently performed combined organ transplantation involving the liver, thereby providing a comprehensive overview of eligibility criteria, allocation systems, surgical procedures, and clinical outcome.

## Multi-organ Allocation Policy in the Eurotransplant Zone and the United States

In the Eurotransplant zone, livers are allocated internationally first to high urgency status patients and then to those with approved combined organ (ACO) status. In contrast, elective liver transplants are offered on a national basis where allocation might be recipient- or center-driven. An ACO status can be requested in need of a liver combined with heart, lung(s), intestine, or pancreas, which will be reviewed by one Eurotransplant Liver and Intestine Advisory Committee member and, depending on the other organ request, by one member of the Eurotransplant Thoracic or Pancreas Advisory Committee. 5 In case of a pediatric donor, pediatric patients with ACO status are prioritized over adult patients. When an offer to an ACO status patient is made, the “leading” organ initiates the match. Eurotransplant offers the organs in a sequential order: heart first, followed by lung(s), liver, intestine, pancreas, and kidney. In case the donor and recipient are not from the same country, an obligation is generated, meaning that the receiving country is obliged to offer the next available liver in the same blood group to the country they received a liver from for their ACO patient. 6 Of note, CLKT is not included in the ACO system. The principle of allocation for CLKT is based on the model for end-stage liver disease (MELD) score and not on the kidney allocation system which primarily considers waiting time.

In the United States, a clear order in which organs should be allocated does not exist. The Organ Procurement Transplant Network (OPTN) released an update of their policies in March 2024. Comparable to Europe, MOT is generally prioritized above single-organ transplantation, but there are no policies prioritizing certain multi-organ combinations above others. When the local organ procurement organization (OPO) offers a heart, and a liver is available from the same donor, the OPO must also offer the liver if the recipient is registered at a transplant hospital at or within 500 nautical miles (NM) of the donor hospital and if the recipient fulfills Heart Adult Status 1, 2, 3 or has any active pediatric status. 7 If the OPO offers a lung, the liver must be offered to the same recipient if a Lung Composite Allocation Score ≥25 is met. When a liver is being offered, and a kidney is available from the same donor, the kidney must be offered to the recipient who is registered for a liver and a kidney at the same transplant hospital and who meets one of the following criteria: (1) the recipient was less than 18 years old when registered on the liver waiting list; (2) the recipient is registered at a transplant hospital at or within 150 NM of the donor hospital and has a MELD ≥15 and meets eligibility criteria for liver–kidney allocation; (3) the recipient is registered at a transplant hospital at or within 500 NM and has a MELD ≥29 and meets eligibility criteria; (4) the recipient is registered at a transplant hospital at or within 500 NM and is adult status 1A and meets eligibility criteria. Eligibility criteria for CLKT have been introduced by the OPTN in 2017. They are also listed in the 2021 American Association for the Study of Liver Diseases guidelines for diagnosis, evaluation, and management of ascites, spontaneous bacterial peritonitis, and hepatorenal syndrome and will be discussed later. Once patients that require CLKT are listed, organs are allocated to that individual based on the liver match sequence. As a consequence, all other kidney-alone transplant candidates including those with high priority such as highly human leukocyte antigen (HLA)-sensitized candidates, pediatric candidates, and prior living donors, are prioritized below candidates for potential CLKT, thereby impacting access to kidney transplantation for vulnerable candidates. 8

In the special case of kidney-after-liver safety net applying to patients with non-recovered kidney function after liver transplantation, candidates are ranked after patient groups with high priority for kidney-alone transplants mentioned earlier, but before other local blood type-compatible adults. 9 For liver transplant recipients who do not recover kidney function 60 to 365 days posttransplant, the safety net set up by the OPTN allows preferential waitlist access for a kidney transplant. In the Eurotransplant zone, this is referred to as the “kidney after other organ bonus.” The eligibility criteria are similar to the OPTN's, with some country-specific variations. Patients fulfilling the criteria will receive 500 bonus points. 10 The safety net intended to limit “prophylactic” kidney transplants in cases with potential for post-liver transplant kidney recovery. It was shown to mitigate morbidity and mortality 11 although it might carry immunological disadvantages. An alternative approach to the safety net, which prioritizes post-liver transplant patients above most local kidney-alone transplant candidates with longer waiting time, would be to promote living kidney donation which should be discussed with the patients prior to liver transplantation. 9

In the case that organs from a single donor could be allocated to several multi-organ candidates with different combinations, the local OPO decides which multi-organ patient receives prioritization, which has the potential to impact equity. 12 Another example where decision is left to the OPO's discretion, and thus potentially interfering with equity, is multivisceral transplants. In case of liver–intestine transplants, the candidates are registered on the intestine and liver waitlists. When a liver is allocated to the candidate based on liver allocation policy, the intestine is also offered from the same donor. However, it is at the OPO's discretion to also allocate any other needed organs such a kidney or pancreas to the multivisceral candidate. 7 13

Specific guidelines and eligibility criteria for organ combinations such as CHLT, CLLT, or liver–intestine do not exist, giving room for controversial debates.

## Combined Heart–Liver Transplantation

### Indications, Eligibility, and Outcome

The first CHLT was performed in 1984 in a 6-year-old girl with severe heart disease due to homozygous familial hypercholesterolemia. 14 CHLT is still an uncommon but increasingly performed procedure, with congenital heart disease (CHD) having surpassed non-CHD as the most common indication. 15 This is mainly attributed to the growing pediatric population with CHD reaching adulthood. 16 Other indications include metabolic diseases which are curable with liver transplantation such as hereditary transthyretin amyloidosis, hemochromatosis, and, as mentioned, familial hypercholesterolemia.

Currently, there are no consensus criteria regarding CHLT, and considerations are focused on whether liver disease is so advanced as to affect perioperative risk of a heart transplant or might reverse with improvement of cardiac function. An algorithm for CHLT evaluation was proposed by the American Heart Association based on published data and expert consensus, 17 and a more specific algorithm for evaluation of patients with CHD and Fontan physiology which eventually leads to Fontan-associated liver disease (FALD) was proposed by Taner et al. 18 Patients considered for CHLT should all undergo hepatic function assessment based on laboratory testing (liver synthesis parameters and liver enzymes), MELD-XI score which is a modified MELD score that excludes international normalized ratio (INR) and abdominal imaging by ultrasound or computed tomography. It was shown that a MELD-XI above 14.1 is associated with increased mortality, infections, stroke, dialysis, and rejection after heart transplant. 19 If the MELD-XI is low (<11), and laboratory parameters and imaging are normal, patients should be considered for heart transplant only. In case of possible or advanced liver disease, liver biopsy with transjugular pressure gradient measurement should be considered. Only patients with biopsy-proven cirrhosis, or severe liver fibrosis with elevated transhepatic pressure gradient, or other signs of portal hypertension should be further evaluated for CHLT. 17 However, numerous uncertainties persist, and decisions are often guided by center-specific guidelines and expertise available within each institution, which has the potential to lead to inequities across transplantation centers.

Analysis of the United Network of Organ Sharing (UNOS) registry data suggests similar survival after CHLT compared to heart transplant alone. 1 20 21 According to these analyses, the 5-year survival for CHLT is reported to be between 74.3 and 81%. 1 21 Also, when specifically assessing outcomes of patients with CHD, no difference in long-term survival after 5 years could be detected between patients with CHD versus no CHD. 22

### Surgical Procedure

Various surgical techniques have been used for simultaneous heart–liver transplantation, ranging from separate to en bloc transplantation. 23 24 Among these, sequential transplantation is the most frequent surgical approach, whereby the organs are procured separately. Heart transplantation is performed on cardiopulmonary bypass (CPB), after which the recipient is weaned from. The chest is left open, which facilitates intra-abdominal exposure and allows immediate connection to mechanical circulatory support if required. Liver transplantation is then performed off-pump or with the use of veno-venous bypass or veno-arterial extracorporeal membrane oxygenation (ECMO), depending on center preference and patient's condition. 23 25 Conversely, in en bloc CHLT, the organs are procured with the connecting vena cava remaining intact: the recipient undergoes cardiectomy and hepatectomy with CPB; the heart and liver are then transplanted simultaneously and reperfused together while on CPB. 26 27 The en bloc technique might be beneficial in patients with FALD to protect the liver from longer cold ischemia time and the heart from metabolic and hemodynamic derangements. 27 In a series of six cases, it was reported that a liver-first strategy was successfully performed in highly sensitized patients to take advantage of the immunoprotective effects of the liver. 28 Hypothermic oxygenated machine perfusion (HOPE) now available for both liver and heart preservation presents a promising tool to optimize transplantation logistics and outcomes particularly in patients with FALD. 29 30

Whichever surgical or preservation technique is used, matching of donor and recipient size is essential to ensure optimal posttransplantation outcomes. In patients undergoing heart transplantation, total predicted heart mass is the recommended metric to assess donor–recipient size matching in order to avoid accepting an undersized donor heart. 31 While oversizing is a preferred means to compensate for elevated pulmonary vascular resistance in heart transplantation, oversizing of the liver may pose technical challenges. Both need to be carefully considered when a combined donor offer is accepted.

### Perioperative Management

In accordance with general heart-transplant guidelines, use of vasopressors and inotropic support should be minimized to avoid potential vasoconstriction in the hepatic allograft. Furthermore, minimization of renal vasoconstriction is crucial for restoration and stabilization of renal function and therefore optimal mid- and long-term outcome. These aspects need to be taken into consideration even during the initial postoperative phase. 32

The postoperative nutrition should be oralized as soon as possible and intravenous nutrition has to be avoided since it carries the risk of infection, fluid overload, and electrolyte imbalance. 33 The postoperative immunosuppression consists of a standard triple immunosuppression with a calcineurin inhibitor (preferably tacrolimus), an antimetabolite (with preference for mycophenolate), and steroids. 34 35 All medications can be administered intravenously, but immunosuppression also should be oralized as soon as possible. In our center, calcineurin inhibitors are administered continuously through the intravenous route and trough levels are measured. Target tacrolimus trough levels during the initial 3 postoperative months should be in the range of 10 to 12 ng/mL. Most centers worldwide do not use a further induction therapy such as antithymocyte globulin.

Most common reasons for early postoperative mortality are infections and cardiovascular mortality. 21 Therefore, standardized surveillance protocols in the initial postoperative period are crucial to achieve optimal survival. Rejection episodes and cardiac allograft vasculopathy (CAV), which occur most commonly in the first 3 years after transplantation, are the main drivers for mid- and long-term mortality. Noninvasive ultrasound-guided surveillance after heart transplantation has many advantages with respect to patient comfort and availability, and is able to rule out relevant rejection episodes and progress of CAV with high sensitivity and therewith reduced need for invasive procedures. 36

## Combined Liver–Lung Transplantation

### Indications and Patient Selection

CLLT is one of the least performed types of MOT. The three principal indications include cystic fibrosis (CF) with a prevalence of cirrhosis in 5 to 15% of cases, 37 38 α1-antitrypsin deficiency, and porto-pulmonary hypertension as a consequence of liver cirrhosis. 39

The International Society for Heart and Lung Transplantation recommended in a consensus paper that patients should meet lung disease-specific criteria for lung transplant listing and display advanced liver disease as defined by histologically proven cirrhosis and a portal gradient >10 mmHg. 40 CF-specific criteria for evaluation are forced expiratory volume in the first second (FEV1) <30%, 6-minute walk distance <400 m, or development of pulmonary hypertension. 40 It is also suggested to not select patients with severe impairment of liver function defined as albumin <2.0 g/dL, INR >1.8, or presence of severe ascites or encephalopathy. 40 At the Hannover Medical School, Germany, patients with CF are usually first evaluated for a lung transplant, and in case of evidence of associated liver disease during pretransplant workup, they undergo further evaluation in the department of hepatology. 41 Patients with advanced liver fibrosis accompanied by portal hypertension or established liver cirrhosis finally qualified for CLLT. 41 Published guidelines regarding CLLT in patients with α1-antitrypsin deficiency are lacking, but it is assumed that patients with advanced cirrhosis might benefit from combined organ transplantation. 39

### Surgical Procedure

Liver-first versus lung-first strategy is still under debate. 42 Surgical techniques for CLLT and lung-only transplant have been previously reported by Grannas et al and Salman et al. 43 44 At the Broussais Hospital, all operations involving patients with CF from 1989 to 1995 were performed on CPB due to the critical nature of the recipient's condition (all patients required supplemental oxygen before undergoing transplantation); liver transplantation was performed after completion of lung implantation and termination of bypass. 45 In recent years, ECMO has become the mainstay of cardiopulmonary support during lung transplantation, reducing the need for anticoagulation and thus bleeding complications and blood transfusions. Reports on successful lung transplantation after extended ischemic time of 10 hours and more and the availability of ex vivo lung perfusion (EVLP) have led to propose a liver-first strategy as opposed to lung-first. 41 Liver-first has the potential benefits of improved coagulopathy and protection of the lung allograft from liver reperfusion fluid. The disadvantage of liver-first is the longer lung cold ischemia time, which might be mitigated by EVLP.

As a side note, Couetil et al suggested to avoid choledochocholedochostomy in patients with CF due to postoperative development of biliary strictures. Accordingly, a Roux-en-Y choledochojejunostomy should be preferred. 45

### Postoperative Management and Outcome

The immunosuppressive treatment most commonly reported is based on the regimen for lung transplant. Maintenance can be often achieved by triple immunosuppressive therapy consisting of calcineurin inhibitors, mycophenolate, and prednisolone. 41 46 Since CLLT recipients often present with chronic colonization, posttransplant antibiotic prophylaxis is recommended. Different center-dependent anti-infective regimens exist. 46 47 The Hannover Medical School in Germany suggests a combination of flucloxacillin and meropenem for 2 weeks and tobramycin for 10 days. Available information on pretransplant bacterial colonization should be taken into account. 41

A retrospective analysis of the UNOS database from 1987 to 2010 which included 122 patients undergoing CLLT found that the 5-year survival for CLLT was 59% and did not differ from lung-only recipients. The long-term survival up to 10 years was comparable between patients with CF who received lung-only transplants and those who underwent CLLT. 41 Lung rejection episodes were more frequent than liver rejection episodes, 45 but overall rejection was not more frequent in patients with CF who received CLLT than in patients with isolated lung transplant. 45 On the contrary, incidence of histologically confirmed lung rejection was significantly lower in patients undergoing CLLT than lung-only recipients. 41

## Combined Liver–Kidney Transplantation

### Indications and Patient Selection

The first successful CLKT was performed 40 years ago in Innsbruck, Austria, in a patient with a failing kidney allograft and hepatitis B-induced liver cirrhosis. 48 Since then, CLKT has evolved as the mainstay of treatment for ESLD who present with chronic kidney disease (CKD), prolonged acute kidney injury (AKI), or metabolic diseases with kidney impairment, based on OPTN's revised CLKT policy implemented in 2017. 49 For CLKT eligibility, CKD is defined as glomerular filtration rate (GFR) <60 mL/min for 90 consecutive days and either GFR ≤30 mL/min or receiving dialysis at the time of registration. On the other hand, metabolic disorders include hyperoxaluria, atypical hemolytic uremic syndrome, familial nonneuropathic systemic amyloidosis, as well as amino and organic acid-related disorders such as propionic and methylmalonic aciduria. 49 50 Thus, patients waitlisted for CLKT represent a highly heterogenous collective, resulting in discrepancies in posttransplant outcomes. 51

In patients with decompensated cirrhosis, severe hemodynamic and immunological changes such as elevated portal venous pressures and pooling, splanchnic arterial vasodilation with concomitant microcirculatory vasoconstriction, and decreased circulating blood volumes are well-recognized causes of renal impairment and most of these cases can be defined as hepatorenal syndrome. 52 Systemic inflammation, acute volume shifts, infections, and diuretics further contribute to AKI, which occurs in approximately a quarter of patients with liver cirrhosis. 53 54 Although initially reversible, hepatorenal syndrome in ESLD has been clearly associated with a drastic increase of both acute and long-term morbidity and mortality. 54 Reflected in the MELD score, which has determined organ allocation for over two decades, patients with ESLD and increased creatinine or requirement of dialysis have preferential waitlist positions for liver transplantation. 55

The central difficulty in the selection of patients with cirrhosis for CLKT with sustained AKI, defined as receiving dialysis and/or persistent GFR ≤25 mL/min for 6 consecutive weeks, instead of liver transplant alone lies in predicting the recovery of kidney function after liver transplantation. 56 For instance, posttransplant kidney function in patients with acute-on-chronic liver failure (ACLF) is significantly worse than after non-ACLF indications, 57 and assessing pretransplant kidney function is hampered by poor performance of creatinine-based GFR-estimating equations. 58 While a large fraction of patients with AKI recovers kidney function after liver transplantation, severe or prolonged posttransplant kidney dysfunction is clearly associated with impaired liver allograft function and overall patient survival due to a higher incidence of sepsis and cardiovascular incidents. 59 Metabolic dysfunction-associated steatotic liver disease (MASLD), emerging as one of the most frequent causes of ESLD worldwide, and diabetes were identified as negative prognostic parameters for delayed postoperative kidney function and stage 4–5 CKD after CLKT in a multicentric U.S. consortium. 60 At the same time, patients with MASLD and preserved kidney function (GFR > 30 mL/minute/1.73 m ^2^ ) undergoing liver transplantation had a reduced risk of all-cause mortality, compared to patients with GFR <30 mL/minute/1.73 m ^2^ undergoing CLKT or liver transplant alone. 61

### Postoperative Management and Clinical Outcome

Immunosuppressive regimens vary between centers, but overall it mirrors that of liver rather than that of kidney transplantation. 62 The most common regimen consists of tacrolimus, steroids, and an antimetabolite. 63 Of note, induction therapy with rabbit antithymocyte globulin is associated with higher mortality in CLKT patients. 64

Retrospective UNOS database analyses demonstrated excellent outcomes of patients who underwent liver transplantation alone in accordance with less liberal kidney function criteria, but now newly qualify for CLKT listing. 51 65 Despite a similar rate of short-term liver allograft loss, a recent meta-analysis found that patients with CLKT had a significantly higher 3-year liver allograft survival. 66 However, in a registry analysis of the United Kingdom, no difference in survival was found between CLKT and liver transplant recipients, and 5-year survival was reported to be 83.7% versus 78.5%. 67 Differences in patient survival in patients undergoing CLKT versus liver transplantation alone for ESLD with concomitant renal dysfunction might be explained by different patient collectives with different cut-offs for kidney function, with some authors describing similar short- and long-term mortality with the caveat of more advanced ESLD in the CLKT group, 66 while others found that patients meeting CLKT allocation criteria had a lower risk of death and graft loss if undergoing CLKT. 68 The leading cause of death in CLKT patients was reported to be malignancies and infections. 69

## Multivisceral Transplantation

Given the unique immunologic characteristics of the intestine, it is one of the most difficult organs to transplant. 70 Patients suffering from short bowel syndrome and intestinal failure face challenges in maintaining their fluid and nutritional equilibrium. This particularly affects individuals with type 1 short bowel syndrome and a high jejunal stoma, necessitating total parenteral nutrition. Within 5 years, approximately 50% of these patients develop intestinal failure-associated liver disease (IFALD), with a higher incidence among children. IFALD often progresses to ESLD, contributing to up to 26% of fatalities during parenteral nutrition. 71 The European Society for Clinical Nutrition and Metabolism defines IFALD as liver injury occurring alongside intestinal failure due to parenteral nutrition, in the absence of primary liver diseases, other hepatotoxic factors, or bile obstruction, without mandating liver histology. 72

Patients with IFALD should be referred to transplant centers for evaluation regarding multivisceral transplantation. Although criteria for liver and intestinal transplantation in IFALD lack global consensus, recent guidelines suggest consideration when bilirubin exceeds 100 µmol/L and when portal hypertension coexists with a thrombocyte count below 150,000/µL. 73 However, long-term survival remains suboptimal, with retrospective studies indicating 1-year survival rates of 80%, dropping to 20% at 5 years posttransplantation. 74 75 Risk factors for high mortality are high blood levels of tacrolimus, the use of large intravenous boluses of prednisone, duration of operation, and transplantation of cytomegalovirus (CMV)-positive organ donor to CMV-negative recipients. 76 Despite these challenges, multivisceral transplantation remains a viable option for select patients, particularly when IFALD remains compensated, potentially reducing the reliance on parenteral nutrition and preventing IFALD progression. 77 Although extended portomesenteric thrombosis may serve as an additional indication for combined transplantation, it is rarely performed and reserved for highly specific cases. 78

## Dual Living Donor Transplants

In general, dual organ transplants can be performed from the same or different living donors and either simultaneously or sequentially. Living donor combinations involving the liver which have been performed include liver–kidney and liver–intestine transplants, and for both combinations simultaneous or sequential organ procurement has been described. The 2005 Vancouver forum emphasized that live donation should only be performed if the combined benefits to the donor–recipient exceed the risks to the donor–recipient pair. 79 Hence, it is understandable that transplantation of two organs from one living donor is a very infrequently performed procedure, and ethical considerations revolving around liver–kidney donation from the same living donor are elegantly presented by Bababekov and Pomfret. 80 The first successful simultaneous liver–kidney transplant (left lateral segments of the liver) from one living donor was performed 1992 in Turkey, and in 1999, the first simultaneous right lobe liver–kidney transplantation was undertaken in Brazil. 81 Around 20 years later, the first case of laparoscopic procurement of partial liver and kidney was described. 82 However, minimally invasive organ procurement for simultaneous donation is limited to highly specialized centers and not standard practice. 83 Common indications in the pediatric cohort for living liver–kidney donation are autosomal-recessive polycystic kidney disease and primary hyperoxaluria type 1. 83 84 The most severe complications among the living donors in these studies were reported to be of biliary nature (e.g. biliary leakage and fistula) and bleeding. Based on a study which analyzed UNOS data spanning four decades till 2022, in the adult population the most frequent indication (80% of cases) of simultaneous liver–kidney transplantation from living donors was cirrhosis of the liver. 85 For sequential liver–kidney transplant (from different living donors), the most common reason was calcineurin toxicity followed by type 2 diabetes mellitus, and the most frequent indication for subsequent liver after kidney transplantation in adults was polycystic kidney disease. 85 Encouragingly, no procurement-related death occurred in the living donors. 85 According to the same study, four simultaneous and three sequential liver–intestine transplants with organs from the same living donor and two sequential liver–intestine transplants from different living donors have been performed in the United States. All recipients were pediatric patients who received organs from related donors. 85 86 87 The main indication was intestinal failure with IFALD. Despite all the advances, it needs to be emphasized that simultaneous and sequential liver–kidney or liver–intestine transplantation still poses a major surgical procedure to the living donor. For now, these procedures are reserved for extreme cases, often involving close relatives for whom no other life-saving options exist. 80

## Innovative Techniques: Organ Preservation Methods

Patient selection, organ shortage, prolonged cold ischemia, and lack of biomarkers predicting recovery of organ function after liver transplantation represent key challenges in combined liver transplantation. Strategies to overcome this include biomarkers to aid patient selection and machine perfusion (MP) to release time constraints and improve graft viability.

Static cold storage (SCS), the conventional preservation and transportation of donor organs on ice, constituted the gold standard in organ transplantation for decades due to its practicality and sufficient preservation of high-quality donor allografts. Expanded indications for organ transplantation and donor shortage led to the increased acceptance of extended criteria donation (ECD) allografts—for example, organs from elderly donors or organs subjected to extended preservation times. 88 While the implantation of ECD allografts reduces wait-list dropout and has acceptable outcomes, 89 the postoperative function of ECD organs is particularly impaired by ischemia-reperfusion injury (IRI) after SCS. 90

MP preoperatively mitigates transportation injury, improves postoperative outcomes, and prolongs preservation times. 91 Several randomized controlled trials have provided high-level evidence of both HOPE and NMP reducing early allograft dysfunction in liver transplantation alone. 30 92 93 94 The underlying mechanism of organ protection during HOPE is that mitochondrial oxygen levels are restored hypothermically, preventing mitochondrial injury during reperfusion. 95 It could be shown in animal models that MP reduces the levels of pro-inflammatory cytokines, protects against epithelial and Kupffer cell activation, and reduces recipient T cell infiltration to the donor graft. 96 97 Kvietkauskas et al recently summarized the immunological aspects of MP. 98

In kidney transplantation, continuous hypothermic MP from retrieval to implantation has decreased the incidence of delayed graft function (DGF) in marginal allografts. 99 At the same time, end-ischemic MP, both HOPE and NMP, failed to demonstrate reproducible benefits in marginal kidney grafts. 100 101

While the rationale for MP in liver transplant is clear—loosening time constraints, reducing IRI, and enabling preimplantation viability assessment 102 103 —the current level of evidence in combined transplantation is limited to retrospective analyses and single-center case series. CLKT presents considerable requirements to surgical logistics and to the intraoperative management of hemodynamics. 49 Significant intraoperative hemodynamic shifts are inherent to liver transplantation, both due to the intraoperative clamping of large vessels, particularly the vena cava, and arterial vasodilation and increased intra-abdominal pressures due to portal hypertension. 104 The intraoperative use of vasopressors and blood loss related to portal hypertension and coagulopathy is far from ideal for the newly implanted kidney allograft. 49 To protect the kidney allograft, Ekser et al in Indianapolis therefore came up with a compelling strategy to delay kidney transplant by preserving kidney grafts for a mean additional 40 hours after liver transplantation with hypothermic pulsatile MP. As a result, DGF was reduced significantly, and long-term patient survival was improved. 105

A UNOS analysis found that in 2019, over 25% of kidney allografts used in combined liver transplantation were machine-perfused. In those allografts, a reduction of DGF, but not primary non-function, was observed, and kidney graft survival was only improved in low-risk allografts. 106 Regarding CHLT, the Mayo Clinic Florida reported three cases of successful ex situ NMP initiated at the donor hospital. 107 Lactate and bile production were monitored for all the pumped livers. The authors argue that NMP allowed them to wait for the patient to be weaned from CPB and to be hemodynamical stable. It also has the potential to lessen metabolic disturbances associated with hepatic reperfusion and therefore stress on the newly implanted cardiac graft. 107 The Henry Ford Hospital in Detroit, Michigan reported that they used NMP successfully in four patients undergoing CLLT. 108 The lung portion was transplanted first, and the median duration of liver NMP was 413 minutes. One patient developed intra-operative post-reperfusion syndrome, but none of the patients developed secondary liver IRI. 108 These reports show that MP is feasible in combined liver transplantation, but randomized controlled trials are needed to substantiate its beneficial aspects in MOT.

## Immunological Advantage of Combined Liver Transplantation—The Science Behind

Potential benefits of MOT include improved survival, avoidance of uncertain waiting time, and possible immunologic protection. The immunoprotection that the liver, which is well known for its tolerogenic and immunoregulatory properties, 109 110 111 might provide to other organs transplanted simultaneously stems from clinical as well as experimental data. T-cell-mediated rejection and antibody-mediated rejection of the kidney allograft in recipients of CLKT are less frequent compared with solitary kidney transplant recipients. 18 112 Therefore, immunological advantages are considered as an important contributor to the excellent long-term graft and patient survival after CLKT. 113 In fact, this phenomenon has been successfully made use of to protect the kidney in a positive cross-match by simultaneously transplanting a partial auxiliary liver from the same donor. 114 There is less wealthy evidence with regard to other liver–organ combinations. However, the findings are in keeping with the observations in CLKT recipients. In a single-center study, early rejection rate of patients receiving CLLT was lower than that of solitary lung transplant. 43 Similarly, patients with CHD receiving CHLT experienced less rejection episodes compared to patients with heart transplant alone, which is mirrored by the lower incidence of cellular rejection and CAV as assessed by coronary three-dimensional volumetric intravascular ultrasound. 115 116 CAV has been proposed to be a process partially induced by immune activation potentiated by increased cellular rejection. 117 Similarly, acute cellular rejection and acute antibody-mediated rejection as well as chronic rejection are less frequent in recipients of liver-inclusive versus liver-exclusive intestinal transplant. 76 118

**Fig. 2 Mechanisms of immunoprotection provided by the liver to other organ allografts.**
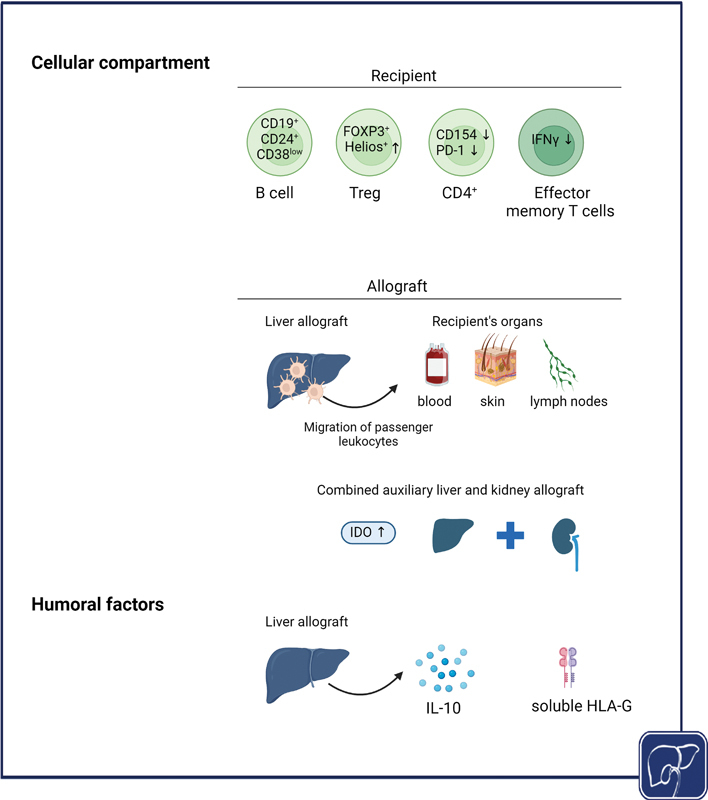
The protective mechanisms comprise cellular as well as humoral components. Combined liver transplantation leads to distinct changes in the circulation of the recipient with preponderance of immune cell populations with immunoregulatory properties. On the other hand, the liver allograft itself induces the release of anti-inflammatory cytokine IL-10 and immunoregulatory HLA-G. Created with BioRender.com. IDO, indoleamine 2,3-dioxygenase; HLA-G, human leukocyte antigen G; IL-10, interleukin-10.

Efforts have been directed towards elucidating the mechanisms behind these observations, which encompass both the cellular and humoral compartments ( Fig. 2 ). Early studies identified the so-called sink effect and chimerism as important mechanisms of immunotolerance. According to the sink effect, donor-specific antibodies (DSAs) are passively absorbed by the endothelial surface of the liver allograft. 62 119 120 121 122 123 124 Preformed DSAs are cleared within 4 months in most CLKT patients, 125 which could partially explain the reduced expression of genes involved in endothelial cell activation and inflammation in kidney biopsies 1 year post-CLKT compared to patients receiving kidney-alone transplants with comparable level of presensitization. 112 Moreover, the liver favors the absorption of HLA class I antibodies. 126 On the other hand, chimerism was first described in 1968 and is characterized by the migration of bone-marrow-derived dendritic cells (DCs), so-called “passenger” donor leukocytes, out of the graft into the recipient's organs. 127 128 129 In mice, these DCs lack immunostimulatory capacity towards allogeneic T cells. 130 There is evidence that migration of “passenger” leukocytes is less in the case of kidney and heart compared to the liver, 131 132 which might explain the higher number of chimeric donor cells in liver than in kidney recipients 133 134 and the higher potential of the liver to confer tolerance. In a rat model, depletion of passenger leukocytes by irradiation of the donor led to graft rejection. 135 However, the concept of microchimerism is controversial since rejection occurs despite the presence of donor-specific microchimerism, and it has been hypothesized that microchimerism is rather a consequence of long-term graft acceptance. 128 136

Another aspect is the unique vascular architecture of the liver with its fenestrated endothelium, absence of basement membrane, and the slow blood flow in the liver sinusoids, which facilitates contact of alloreactive T cells with the tolerogenic environment of the liver, which leads to their deletion. 137 138

It has also been proposed that a certain threshold number of cells is required for graft rejection, and that larger grafts are more resistant to rejection by exhausting the immune response. 119 It has been hypothesized that this effect is not liver-specific but related to antigen load, 139 and could explain the lower incidence of cardiac rejection in recipients of combined heart–kidney grafts compared to heart alone. 140 141 As a proof of concept, it could be shown that liver-specific expression of high doses of donor major histocompatibility complexes (MHCs) in mice conferred protection to skin grafts. 142

Early in the 1980s, soluble factors which confer immunoprotection were put forward: the liver releases soluble class I antigens which neutralize pre-existing alloantibodies. 143 144 Later on, HLA-G, a nonclassical MHC class I molecule which inhibits antigen-specific CD8 ^+^ T cell cytolytic function and induces apoptosis of activated CD8 ^+^ T cells by binding to inhibitory receptors of immune cells, was significantly elevated in the blood of patients with CLKT compared to those with kidney-alone recipients and healthy individuals. 145 Furthermore, none of the patients with CLKT who had HLA-G expression in biliary epithelial cells, as determined by immunohistochemistry of liver graft biopsies, experienced liver or kidney rejection. 145 It has also been shown that liver transplantation as well as combined auxiliary liver–kidney transplantation induces an immediate increase in plasma interleukin (IL)-10, to a much higher extent than witnessed in kidney-alone recipients, which remained high until the subsequent kidney reperfusion. 146 The same group could show that monocyte-derived DCs produced less chemokines such as CXCL9, CXCL10, and CXCL11 and had a reduced capacity of eliciting interferon-Y (IFN-Y) production from T cells when treated with IL-10 in vitro. Interestingly, indoleamine 2,3-dioxygenase, an important immune regulator, is increased in both the liver and the kidney graft in the context of auxiliary liver transplantation. 147

More recent studies have evolved our knowledge on the mechanisms behind the immunomodulatory effect of the liver. They include reduced frequency of activated CD4 ^+^ T cells and donor-specific T cell hyporesponsiveness, 113 higher frequency of CD19 ^+^ CD24 ^+^ CD38 ^low^ memory B cells and FOXP3 ^+^ Helios ^+^ Tregs after CLKT, 148 which are associated with a tolerogenic profile. FOXP3 ^+^ Helios ^+^ Tregs are reported to exhibit greater transforming growth factor β expression and lower cytokine production such as IFN-ү, IL17A, and IL2. 149 150 151 Furthermore, kidneys after CLKT express lower levels of genes associated with inflammation and endothelial cell activation and higher levels of genes associated with tissue integrity. 112

To sum up, the protective effect of the liver is related to the function of the allograft, as the immunoprotective effect is lost in patients with recurrent fibrosis and cirrhosis. Further studies need to clarify where this education of the immune system takes place, whether it is only in the liver or also in the combined allograft, and how it can be integrated with the humoral mechanisms.

## Conclusion

Modifications to current allocation policies are imperative to enhance equity and utility in MOT. With organ scarcity being a persistent challenge, allocating multiple organs to a single recipient raises ethical dilemmas, especially when several individuals could have benefited from those organs. 9 In deliberations about multi-organ allocation, a distinction is often made between “life-saving” and “life-enhancing” transplants. This differentiation underscores the ethical complexities inherent in allocation decisions which are further complicated by the increasing age of donors.

The effect of increasingly performed CLKT on the kidney allograft pool is an important point of consideration 152 : almost half of the kidneys utilized for CLKT in the UNOS region are high-quality allografts that are consequently not available for patients on the kidney waitlist. 56 Balancing the principles of equity and utility becomes particularly complex in such scenarios. Consequently, there is a pressing need for (1) increased research efforts aimed at preventing pretransplant, peri-transplant, and posttransplant kidney dysfunction, (2) refinement of eligibility criteria for combined liver transplantation, and (3) establishing the degree of priority that MOT candidates should receive over single-organ candidates, thereby potentially reducing the necessity for MOT.

To enhance transplantation outcomes, there is a growing interest in employing biomarkers to assess kidney and liver function and predict recovery following heart transplantation. 17 Furthermore, exploring the feasibility of a “liver-after-heart” safety, net policy remains an uncharted territory. The possibility of hepatic decompensation after heart transplantation indicates significant medical risks and underscores the importance of thorough evaluation before implementing such policies. 17

In conclusion, navigating the intricacies of MOT allocation requires a delicate balance between ethical considerations, patient outcomes, and resource utilization. Continued research and dialogue are essential to refine allocation policies and optimize transplantation practices for the benefit of patients in need.

## Notes

Lay Summary Combined transplantation of the liver with other organs such as heart, lung, kidney, and intestine is a technically challenging procedure with the potential to cure certain inherited diseases or to prolong survival in patients with advanced chronic diseases. Here, we discuss how often these different combined transplantations are performed, for which medical reasons, and how patients are selected. We also explain the unique ability of the liver to protect itself and other transplanted organs from rejection.
